# Approach to patients with refractory coeliac disease

**DOI:** 10.12688/f1000research.9051.1

**Published:** 2016-10-20

**Authors:** Ikram Nasr, Iman Nasr, Hannah Campling, Paul J. Ciclitira

**Affiliations:** 1 Gastroenterology Department, St Thomas’ Hospital, London, UK; 2 Department of Allergy and Immunology, Royal Hospital, Muscat, Oman; 3 Kings College London, Medical School, London, UK

**Keywords:** RCD type 1, RCD type 2, ulcerative jejunitis, enteropathy-associated T-cell lymphoma, EATL, non-responsive coeliac disease, celiac disease

## Abstract

Refractory coeliac disease (RCD) is a recognised complication, albeit very rare, of coeliac disease (CD). This condition is described when individuals with CD continue to experience enteropathy and subsequent or ongoing malabsorption despite strict adherence to a diet devoid of gluten for at least 12 months and when all other causes mimicking this condition are excluded. Depending on the T-cell morphology and T-cell receptor (TCR) clonality at the β/γ loci, RCD can be subdivided into type 1 (normal intra-epithelial lymphocyte morphology, polyclonal TCR population) and type 2 (aberrant IELs with clonal TCR). It is important to differentiate between the two types as type 1 has an 80% survival rate and is managed with strict nutritional and pharmacological management. RCD type 2 on the other hand has a 5-year mortality of 50% and can be complicated by ulcerative jejunitis or enteropathy-associated T-cell lymphoma (EATL). Management of RCD type 2 has challenged many experts, and different treatment approaches have been adopted with variable results. Some of these treatments include immunomodulation with azathioprine and steroids, methotrexate, cyclosporine, alemtuzumab (an anti CD-52 monoclonal antibody), and cladribine or fludarabine sometimes with autologous stem cell transplantation. In this article, we summarise the management approach to patients with RCD type 2.

## Introduction

Coeliac disease (CD) is an autoimmune response triggered by dietary exposure to gluten in those individuals who are genetically predisposed ^1^. It results in both gastrointestinal and extra-gastrointestinal symptoms. Typically, patients have diarrhoea, bloating, weight loss, and anaemia. It is a lifelong condition with a prevalence of around 1% in Europe and the US. The gold standard for diagnosis requires a duodenal biopsy showing villous atrophy and increased intra-epithelial lymphocytes ^2^. The only accepted treatment for CD is to completely remove gluten from the diet; this includes cereal, wheat, rye, and barley. Most cases of CD report symptom improvement within a few weeks of a strict gluten-free diet (GFD). Those who do not respond to this diet are said to have non-responsive CD (NRCD). The difficulty of maintaining gluten elimination means that the majority of NRCD is due to continued ingestion. An alternative diagnosis should be sought in those members of the NRCD group whose continuing symptoms appear to be unrelated to CD. An even smaller group are those with refractory CD (RCD), in which a GFD is being adhered to but the symptoms of malabsorption remain. On biopsy, they will have persistent villous atrophy despite at least 12 months on a GFD. RCD can be classified as primary (no response to GFD) or, more commonly, secondary (an initial response to GFD, followed by relapse). RCD can also be classified by its clonality of T-cell receptor (TCR) into RCD types 1 and 2. It is important to make a distinction between RCD types, as type 2 is associated with complications such as ulcerative jejunitis and enteropathy-associated T-cell lymphoma (EATL), giving it a poor prognosis. Identifying the different types of RCD is a complicated process involving polymerase chain reaction (PCR) to determine TCR clonality, histology of the small intestine, and intra-epithelial lymphocyte (IEL) phenotype and morphology ( Table 1) ^3^.

**Table 1.** Comparison between refractory coeliac disease types 1 and 2, ulcerative jejunitis, and enteropathy-associated T-cell lymphoma.

Investigation	Refractory coeliac disease		Ulcerative jejunitis	Enteropathy-associated T-cell lymphoma
	Type 1	Type 2		
Intra-epithelial lymphocyte (IEL) phenotype	More than 70% of IELs are surface CD3 ^+^ and CD8 ^+^	Majority have an aberrant IEL CD3 ^+^/CD8 ^−^ phenotype and rarely have normal CD3 ^+^ and CD8 ^+^	Mucosal ulceration with villous atrophy and IEL in adjacent mucosa	Neoplastic cells are CD3 ^+^ and large cell variants are CD30 ^+^; background IELs are mostly phenotypically abnormal (CD3 ^+^/CD8 ^−^)
Histopathology	Identical to any Marsh classification of coeliac disease	Marsh ≥ II	Mucosal ulceration with villous atrophy and IEL in adjacent mucosa	Infiltration of medium- sized or large pleomorphic lymphoid cells
T-cell receptor gamma gene rearrangement polymerase chain reaction	Polyclonal	Monoclonal	Monoclonal	Monoclonal

RCD type 2 is rare and therefore there are only a few randomised controlled trials to give us treatment recommendations. In some centres, a combination of prednisolone with a thiopurine has been used and shown to give good results for type 1 and variable results for type 2; a clinical improvement was reported in up to 75% of patients with RCD type 2 ^4^. A review by Malamut *et al*. looked at treatment with methotrexate or anti-tumour necrosis factor-alpha in 14 patients with RCD type 1 and 43 patients with RCD type 2 and found that some had a histological response to this treatment ^5^. There have been statistically significant improved survival rates following the use of cladribine (2-chlorodeoxyadenosine, or 2-CdA), whereas the use of alemtuzumab (anti-CD-52 monoclonal antibody) has shown only marginal success ^6^. Previously, the use of azathioprine and prednisolone was associated with progression to EATL, but Nasr *et al*. showed that the use of these drugs in combination was not only successful but also safe, especially where the cases of RCD type 2 were identified early ^3^. The results showed 53% histological recovery and 56% transformation from oligoclonal to polyclonal gamma receptor population, and none of the patients developed EATL.

## Non-responsive coeliac disease

NRCD requires clinical and histological diagnosis. Following 1 year of a strict GFD, these patients will still be complaining of symptoms like abdominal pain, diarrhoea, and tiredness. Blood tests may show low levels of iron, B _12_, and folate, and small bowel biopsy will reveal villous atrophy ^7^. The main objective for managing these patients is to find the cause of the NRCD. Those who are diagnosed with NRCD can be classified into one of four groups on the basis of their history, current symptoms, and investigations: (1) patients who are not adhering to the GFD, (2) patients whose symptoms mimic untreated CD but who in fact have a second diagnosis other than CD, (3) patients with CD and complications, and (4) patients with RCD, which comprises 0.1% to 1% of patients with CD ^3, 8^.

Over 90% of NRCD cases fall into the first group, consisting of those who continue to ingest gluten either by choice or unintentionally ^9^. Nevertheless, when someone presents with NRCD, it is crucial to exclude other diseases which can mimic CD; these include pancreatic insufficiency, lactose intolerance, small bowel bacterial overgrowth, inflammatory bowel disease, hypo-gammaglobulinaemia, tropical sprue, collagenous colitis, and adult-onset autoimmune enteropathy. A detailed investigation is required to rule out these conditions; firstly, it is necessary to make sure the patient is strictly adhering to the GFD. If there are raised CD serology antibody titres, then it can be assumed that there is still ingestion of gluten. Investigations include an upper gastrointestinal endoscopy with biopsy of the small intestine for light microscopy, coeliac serology including IgA antibodies to tissue transglutaminase (tTG) and endomysium and PCR for TCR monoclonality. Immunoglobulin levels, including IgA and IgG titres, are tested by some centres, followed by testing for IgG antibodies to tTG if there is IgA deficiency. Further testing includes HLADQ2 and DQ8 status, colonoscopy, and testing for lactose and fructose intolerance, small bowel bacterial overgrowth, and pancreatic insufficiency. Following this, if CD is confirmed and other causes for the continuing symptoms have been ruled out, a strict GFD must be adhered to and RCD can be considered as the diagnosis.

## Refractory coeliac disease

RCD represents a small subset of the NRCD group who continue to have symptoms of malabsorption and have villous atrophy on small bowel biopsy despite not having ingested gluten for at least 1 year ^10^. This subset of individuals will already have been investigated for other causes, as detailed above, and usually nothing else is found. The differentiation into primary and secondary RCD can then be made according to the onset of symptoms. Those with primary RCD will never have had a response to a GFD, whereas those with secondary RCD will have been initially responsive to dietary elimination of gluten but are now experiencing a return of symptoms. There is no time frame for this; some patients develop secondary RCD decades later. Classifying RCD as type 1 or 2 depends on the phenotype of the IELs. Type 1 has a normal IEL phenotype, and type 2 has an abnormal clonal population with loss of CD8 and expression of intra-cytoplasmic CD3 by IELs. Making this distinction between types 1 and 2 is key in being able to manage RCD appropriately. It also aids with prognosis, as RCD type 2 has a 5-year mortality of 55% compared with just 7% in RCD type 1 ^4^. Ulcerative jejunitis and EATL are the main complications that can occur in RCD, and EATL is responsible for the majority of deaths in patients with RCD. There is a 3:1 female-to-male ratio with RCD type 2, but this is reversed in EATL to result in males being more affected ^11^. HLA DQ2 homozygosity is a risk factor for both RCD type 2 and EATL ^12^.

## Diagnosis

It can be difficult to diagnose RCD. The process involves clinical assessment of the patient. Pathological, histological, laboratory, and radiological findings can all help, but essentially it is a diagnosis of exclusion in many cases. Nasr *et al*. ^3^ describe the strategy used in a tertiary centre for diagnosing RCD type 2:

1. Patients must be on a strict GFD with a dietary assessment of compliance. The tTG/endomysial antibody serology is frequently negative in RCD type 2.
2. Marsh scoring of small bowel biopsy should be obtained during upper gastrointestinal endoscopy.
3. Assessment of IEL phenotyping and PCR for TCR monoclonality at the β or γ loci or both should be carried out. Abnormal (clonal) IEL in the small bowel is supportive of an RCD type 2 diagnosis. Transient TCR clonality can be detected in patients at the time of diagnosis and in those with poor compliance.
4. Capsule endoscopy should be performed in all cases of RCD type 2 to exclude EATL. However, if there is any suspicion of EATL, magnetic resonance imaging (MRI) must be performed before capsule endoscopy to exclude an obstructing lesion. Capsule endoscopy should be repeated 1 year later to check for the development of EATL. Some have suggested that RCD type 2 be renamed pre-EATL.
5. If EATL is suspected, such as when a patient presents with abdominal pain, weight loss, or malnutrition, then cross-sectional imaging, including small bowel MRI, computed tomography scan, and positron emission tomography scan, are recommended. This allows identification of abnormalities within the bowel, abnormal lymph nodes, and involvement of other organs.

## Management of refractory coeliac disease type 2

There have been a variety of attempts to manage RCD type 2. A good clinical response has been seen when using budesonide for RCD, but there is not a clear effect on prognosis ^13^. Nasr *et al*. ^3^ found good results when combining prednisolone with a thiopurine, including azathioprine, mercaptopurine, or thioguanine, and summarised the other trials undertaken in different centres ( Table 2) ^3, 6, 12– 23^. However, the positive results from thiopurines and prednisolone are not echoed by everyone. The Mulder group reported no improvement and/or progression to EATL when treating patients with the same regimen ^4^. Nasr *et al*. argue that their good results are affected by early detection of RCD type 2, close monitoring of patients, adherence to the treatment, and a multidisciplinary approach which uses dieticians and a histopathologist who understands RCD and the availability of PCR ^3^. They also acknowledge that the percentages of aberrant IEL and clonal TCR population may have an effect on response to treatment. Other treatments include the following:

• Methotrexate: used as a single agent or with cyclosporine. There have been a few cases with good results
• Alemtuzumab (anti-CD-52 monoclonal antibody): this has limited data and outcomes
• Cladribine: Mulder *et al*. have used this in 32 patients and 18 of those patients had a good response ^16^
• Cyclosporine: Wahab *et al*. report a 61% histological improvement with the use of cyclosporine to treat 13 patients with RCD type 2 ^21^
• Infliximab has good results reported by single cases, although some of these are for patients with RCD type 1; there needs to be a much larger study of this drug before its value in treating RCD type 2 can be properly established

**Table 2.** Treatment options in refractory coeliac disease type 2.

Management	Outcome
Alemtuzumab (anti-CD-52 monoclonal antibody): 30 mg twice a week for 12 weeks	Treatment was not effective and the patient demonstrated persistent villous atrophy and an increase in aberrant intra-epithelial lymphocytes ^14^
	Treatment was effective in the case report; however, the authors mention γδ T cells but not the aberrant T cell population which determines the risk of enteropathy-associated T-cell lymphoma ^6^
Budesonide: 9 mg (range 6–12 mg)	This provided good clinical response. The authors report budesonide was also used in the maintenance of clinical remission in collagenous colitis ^13^
Cladribine: 0.1 mg/kg per day for 5 days	A total of 32 patients received cladribine and 18 of them had a good response ^15^
	Six of 17 patients had clinical and histological improvement. Clinical improvement was seen in 36% of cases, histological improvement in 59%, and a significant decrease in the number of clonal intra-epithelial lymphocytes in 35%. However, up to 41% developed enteropathy- associated T-cell lymphoma and died despite cladribine therapy ^16^.
Combination of pentostatin (4 mg/m ^2^ every 2 weeks for 24 weeks) and budesonide	Clinical and histological response as well as a decrease but not disappearance of clonal intra-epithelial lymphocytes in one case ^17^
Cyclosporine A: 5 mg/kg per day	Case report of histological and clinical improvement in a 45-year-old woman with refractory coeliac disease type 2 ^18^
	Single cases reported to show improvement of clinical parameters and mucosal abnormalities during treatment with cyclosporine ^19, 20^
	61% showed histological improvement with this treatment in a group of 13 patients with RCD type 2 ^21^
High-dose chemotherapy followed by autologous stem-cell transplantation has been explored for refractory coeliac disease type 2 in a pilot study from a single centre	All seven patients had a significant reduction in the aberrant T cells in duodenal biopsies associated with improvement, but one out of the seven died of progressive neurosyphilis ^22^
	Out of the four patients with enteropathy-associated T-cell lymphoma, one patient sustained remission 32 months after autologous stem-cell transplantation. Three patients died from relapse within a few months after autologous stem-cell transplantation ^23^.
Thiopurine, including azathioprine: 2–2.5 mg/kg per day, mercaptopurine: 1 mg/kg per day, or thioguanine: 20 mg/day combined with prednisolone	52% progressed to enteropathy-associated T-cell lymphoma within 4–6 years ^12^
	The duration of treatment studied was between 12 and 78 months; 60% of the patients transformed to either refractory coeliac disease type 1 or responsive coeliac disease ^3^. 0% progressed to enteropathy-associated T-cell lymphoma.

## Conclusions

RCD is a rare but serious condition. Although relatively high numbers of patients present with continuing symptoms of CD despite following a GFD, the majority of them can be attributed to the NRCD group 1, consisting of those who continue to ingest gluten. It is extremely important to be thorough in history taking, examination, and investigations of those presenting with NRCD in order to identify the small group with RCD. Once RCD has been diagnosed, the process of classification into type 1 or 2 can be undertaken by using small bowel biopsy, PCR, IEL phenotyping, capsule endoscopy, and imaging. Knowing whether a patient has RCD type 1 or 2 gives the opportunity to estimate the prognosis more accurately and guides treatment options.

Various treatment methods have been used in different centres, and the treatment outcome is variable. RCD type 2 is not common; however, early diagnosis and treatment may play an important role in the prognosis. Future areas for research may help identify other treatment options, particularly for difficult-to-treat cases or cases that have progressed.
